# Modeling the Hemodynamic Impact of Y-incision Aortic Annular Enlargements on Aortic Valve Replacement and Valve-in-Valve Procedures

**DOI:** 10.1007/s12265-025-10634-x

**Published:** 2025-06-11

**Authors:** Mia Bonini, Surya Sanjay, Maximilian Balmus, Alexander Makkinejad, Katelyn Monaghan, Marc Hirschvogel, Nicholas Burris, Bo Yang, David Nordsletten

**Affiliations:** 1 Department of Biomedical Engineering, University of Michigan, Ann Arbor, MI USA; 2 Department of Biomedical Engineering and Imaging Sciences, King’s College London, London, UK; 3 Department of Cardiac Surgery, Michigan Medicine, Ann Arbor, MI USA; 4 MOX, Dipartimento di Matematica, Politecnico di Milano, Milan, Italy; 5 Department of Radiology, Michigan Medicine, Ann Arbor, MI USA

**Keywords:** Y-incision aortic annular enlargement, Aortic valve replacement, Computational fluid dynamics, Valve-in-valve, Blood stasis

## Abstract

**Abstract:**

Y-incision aortic annular enlargement (Y-AAE) with surgical aortic valve replacement (SAVR) may improve outflow tract hemodynamics and valve-in-valve (ViV) outcomes but could increase thrombosis risk. We used computational fluid dynamics to analyze post-operative hemodynamics in 15 patient-specific SAVR models, comparing cases with and without Y-AAE. ViV scenarios were simulated by virtually deploying transcatheter aortic valves. Transvalvular peak velocities, pressure gradients, and blood residence time (a proxy for hemostatic risk) were analyzed to assess performance across cases. Y-AAE reduced peak velocity by 39.3% (55% in ViV), transvalvular pressure gradient by 87.2% (92% in ViV), and mean blood residence time by 10.3% (14% in ViV), with no consistent difference in maximum residence time. SAVR with Y-AAE demonstrated improved hemodynamics, even with ViV procedures, and no evidence of increased thrombosis risk.

**Graphical abstract:**

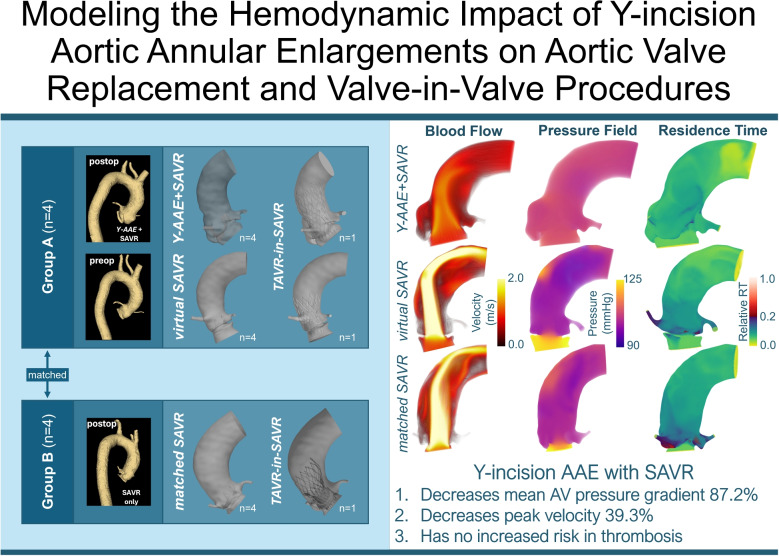

**Supplementary Information:**

The online version contains supplementary material available at 10.1007/s12265-025-10634-x.

## Introduction

**Table 1:** Demographics and Preoperative Data

Variable	*Y-AAE*+SAVR (n=4)	*matched*SAVR (n=4)
	*virtual*SAVR (n=4)	
Age, y	66 (65, 68)	64 (61, 66)
Female Sex	3 (75)	3 (75)
Body Surface Area, m$^{2}$	2.2 (2.2, 2.3)	2 (1.9, 2.0)
Body Mass Index, kg/m$^{2}$	38 (33, 42)	32 (31, 35)
Creatinine, mg/dL	0.7 (0.6, 0.8)	1.2 (0.9, 1.3)
Hypertension	2 (50)	4 (100)
Hyperlipidemia	1 (25)	3 (75)
Diabetes	0 (0)	2 (50)
Bicuspid aortic valve	2 (50)	2 (50)
Previous cardiac surgery	0 (0)	0 (0)
Left ventricle ejection fraction, %	63 (59, 65)	65 (63, 68)
Native annulus size	23 (22.5, 23.5)	22 (21, 24)
Implanted valve size	29 (28.5, 29)	23 (22.5, 23.5)

Data is presented as median (interquartile range) for continuous variables and N (%) for categorical variables. N indicates the number of positive cases, and % indicates the percentage of positive cases over the whole cohort

Aortic stenosis (AS) is the second most common valvular condition in the United States, affecting 5% of adults over the age of 65 [1]. The standard treatment for patients with severe AS is surgical aortic valve replacement (SAVR) [2, 3]. While the advent of SAVR has been transformational in treating AS, challenges remain. In 54-65% of cases, SAVR results in a patient-prosthesis mismatch (PPM), leading to increased transvalvular pressure gradients (TPG), left ventricular hypertrophy, and potential diastolic heart failure [4, 5]. Furthermore, bioprosthetic valve degeneration can occur within 10-20 years requiring intervention [6]. The TAVR-in-SAVR procedure is a non-invasive option to treat valve degeneration; however, PPM remains a serious concern [6]. A strategy to reduce TPG is the Y-incision aortic annular enlargement (Y-AAE), which increases the surgical aortic annulus size. This allows for the implantation of larger valves during the initial operation and larger transcatheter valves in a reoperation if the primary valve replacement fails [7]. However, it is unclear if the larger sinuses could cause abnormal flow, such as decreased sinus velocities, leading to an increased risk of blood stasis and, further, thrombosis [8, 9]. Furthermore, it remains a challenge to use standard clinical metrics (echocardiograms, computed tomography angiography, etc.) to truly isolate how the Y-AAE procedure impacts flow, quantify the thrombosis risk, and compare it to SAVR-only outcomes.

Patient-specific computational fluid dynamics (CFD) is a powerful tool that can simulate blood flow in the cardiovascular system to estimate in vivo metrics that are difficult or impossible to acquire clinically [10]. CFD studies can leverage pre- and post-operative computed tomography (CT) images to achieve patient-specific anatomies for analysis. Computational modeling also allows for the prediction of surgical outcomes by virtually deploying a graft into preoperative geometry [11]. The impact of anatomy can be isolated by prescribing the same boundary conditions between models [12]. Furthermore, CFD modeling allows calculation of complicated flow patterns and metrics including residence time (RT), the time during which a group of fluid particles remains within certain bounds. This is often used as a proxy for measuring the degree of flow recirculation [13].

This study aimed to computationally model hemodynamics in 12 aortic root anatomies, compiled into four groups of patient-specific models of the aortic root with and without a Y-AAE. Additionally, to explore potential valve-in-valve outcomes, we adapted 3 models to include a virtually deployed TAVR. Peak velocities, mean transvalvular pressure gradients, and blood residence times were computed and compared across all model cases. We hypothesized that SAVR with Y-AAE had superior hemodynamics, even when a transcatheter aortic valve was implanted, without increasing the risk of thrombosis.

## Methods

### Medical Imaging and Patient Data

Fifteen distinct models (12 SAVR and 3 SAVR-in-TAVR) were generated. To form our patient cohort, we first reviewed patients undergoing first-time Magna Ease SAVR, with and without Y-AAE, at the University of Michigan Hospital. We included adult patients with severe aortic stenosis and normal aortic annulus size (19-25 mm) that had available pre- and post-operative CT images with contrast. We excluded patients with concomitant ascending/arch replacement (Table 1). From this list we identified 4 patients with SAVR and root enlargement (*Y-AAE*+SAVR). Pre-operative anatomies for these patients were used to virtually deploy a SAVR valve without enlargement (*virtual*AVR) as a control. In addition, each of the 4 *Y-AAE*+SAVR patients were paired to SAVR only patients (n$=$4, *matched*SAVR) from the list that had similar age, sex, body surface area, ejection fraction, and native annulus diameter (Table S1). Finally, a collection of matched *Y-AAE*+SAVR, *virtual*SAVR and *matched*SAVR were used to virtually deploy a TAVR to evaluate valve-in-valve hemodynamics. All patients had pre- and post-procedure CT images acquired on 64-detector CT scanners using helical acquisition mode (GE Medical System Discovery CT750 HD) with a spatial resolution of 0.3 to 0.7 mm. Images were acquired during intravenous iopamidol injection, using retrospective ECG-gating. Institutional review board approval was obtained (protocol No. HUM00211344), with no informed consent required.

**Fig. 1:**
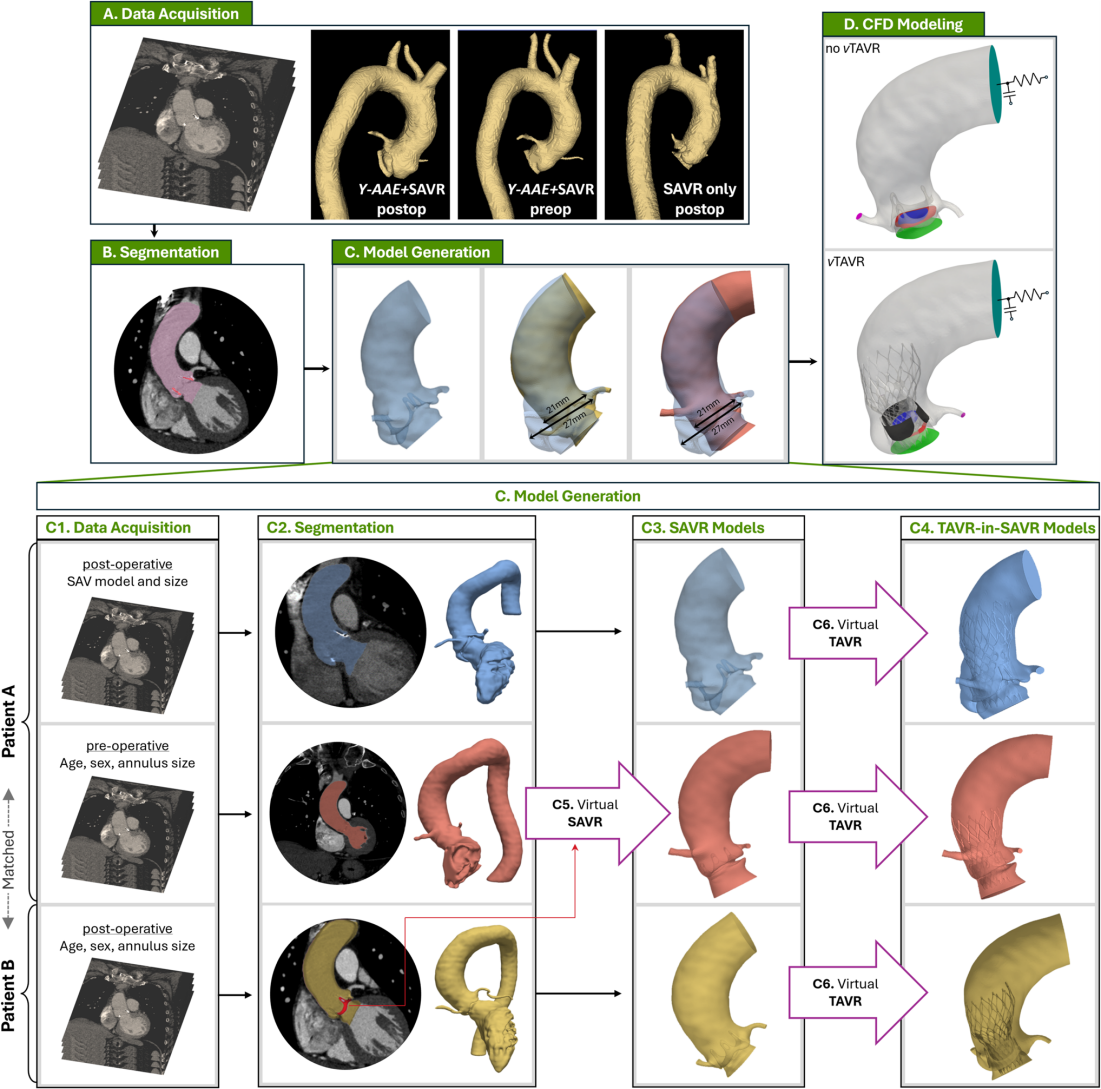
Modeling Pipeline and Model Generation: (A) Pre- and post-operative data acquisition, (B) segmentation of aortic root blood volume and SAVR, (C) model generation, and (D) CFD modeling where flow is prescribed at the inflow plane (green) during systole and pressure during diastole. Valve plane (blue) is open during systole and closed during diastole. SAVR valve leaflets (black), SAVR and TAVR stent (gray). A two-element Windkessel model is prescribed at the outflow plane (cyan), and volumetric flux is prescribed at the coronary arteries (magenta). (C) Model generation for the *Y-AAE*+AVR (row 1), *virtual*AVR (row 2), and *matched*AVR (row 3). To that end, C1) data is acquired from two patients, and C2) segmented to generate C3) the final SAVR models. A virtual TAVR deployment is performed to generate TAVR-in-SAVR models (C4). (SAVR, surgical aortic valve replacement; TAVR, transcatheter aortic valve replacement; Y-AAE, Y-incision aortic annular enlargement)

### Anatomic Reconstruction and Model Generation

We aimed to evaluate the hemodynamic impact of Y-AAE on SAVR procedures. This was achieved by reconstructing patient-specific anatomies from axial CT images and generating computational models (Fig. 1). The process began with the manual segmentation of CT images which were then converted into tetrahedral volume meshes with boundary layers using SimModeler software [14]. Each model had an average element side length of 1 mm and boundary layer edge length of 0.15 mm.

The model generation pipeline is illustrated in Fig. 1C. Post-operative CT images from a patient who underwent SAVR with Y-AAE (‘Patient A’) were used to create the *Y-AAE*+SAVR models (Fig. 1C, top row). Patient B, who underwent SAVR without Y-AAE, was selected as a control, matched to Patient A using parameters from Table S1 (*matched*SAVR). Additionally, pre-operative CT images of Patient A were utilized to build an additional control model for comparison with the *Y-AAE*+SAVR. To generate this control, a Carpentier-Edwards PERIMOUNT Magna Ease aortic valve was virtually implanted into Patient A’s pre-operative anatomy (Fig. 1C5), creating a 3D anatomical model representing post-SAVR without Y-AAE (*virtual*AVR). A 3D model of the Magna Ease valve from the *matched*SAVR patient (‘Patient B’; Fig. 1C, bottom row) was virtually implanted and subtracted from Patient A’s pre-operative 3D model, leaving only the blood volume for the *virtual*AVR.

To study the impact of Y-AAE in potential TAVR-in-SAVR, we virtually deployed (Fig. 1C6) a Medtronic CoreValve into the anatomies of *Y-AAE*+SAVR, *virtual*SAVR, and *matched*SAVR (Group 3; see Fig. 1C4). To create the TAVR-in-SAVR models, a 3D model of a CoreValve was resized based on the primary implanted Magna Ease valve size and Medtronic guidelines [15]. Commissures of the two valves were aligned and the bottom of the CoreValve was positioned to be 4 mm below the bottom of the Magna Ease valve [16, 17]. The Magna Ease leaflets were held in an open configuration (Fig 1D, *v*TAVR).

### Computational Fluid Dynamic Modeling

A stabilized form of Navier-Stokes equations was used to solve for the blood flow (pressure and velocity) in the different anatomies [18]. Blood was modeled as an incompressible, Newtonian fluid. To predict the flow, we utilize the finite-element-based solver $\varvec{\mathcal {C}}$ **Heart** to perform CFD simulations [19]. The pressure (p) and blood velocity (${\textbf {v}}$) are determined by solving the Navier-Stokes equations within the given domain,$\Omega$. These equations read:

1 $$\begin{aligned}&\rho \frac{\partial {\textbf {v}}}{\partial t} + \rho {\textbf {v}} \cdot \nabla {\textbf {v}} - \nabla \cdot \varvec{\sigma } = \varvec{0}, \,\qquad \qquad \qquad \text {on } \Omega _t, \end{aligned}$$

2 $$\begin{aligned}&\nabla \cdot {\textbf {v}} = 0, \qquad \qquad \qquad \qquad \qquad \qquad \qquad \qquad \text {on } \Omega _t, \end{aligned}$$

3 $$\begin{aligned}&\varvec{\sigma } \cdot \hat{{\textbf {n}}} = \varvec{t}_N, \qquad \qquad \qquad \qquad \qquad \qquad \qquad \quad \, \text {on } \Gamma _t^N, \end{aligned}$$

4 $$\begin{aligned}&{\textbf {v}} = {\textbf {{\textbf {v}}}}_D, \qquad \qquad \qquad \qquad \qquad \qquad \qquad \qquad \quad \text {on } \Gamma _t^D, \end{aligned}$$

where $\rho$=1.026 g/ml is the fluid density, $\varvec{\sigma } = \mu _f(\nabla {\textbf {v}} + \nabla {\textbf {v}}^T)+ p{\textbf {I}}$, is the Cauchy stress tensor, $\Gamma$ is the boundary on $\Omega$, and $\mu _f$ =0.004 Pa$\cdot$s is the bulk fluid viscosity. A stabilization scheme proposed by Hoffmann et al. was applied allowing for $\mathbb {P}^1-\mathbb {P}^1$ elements to be used for fluid velocity and pressure without causing spurious oscillations [20].

The aorta was modeled as a rigid domain with no-slip conditions at the wall. The aortic valve was modeled as an orifice plane, opening and closing at the beginning and end of systole (Fig. 1D, blue) [21]. While this simplification neglects valvular dynamics, this approach models the “best-case scenario” as leaflet dynamics tend to increase maximal valvular velocity and increase mean TPG. To model the true aortic valve internal geometric orifice area (GOA), it was prescribed to be 6.0 mm less than the implanted valve’s internal diameter [22]. To isolate the impact of anatomy and facilitate comparisons across the cohort, we prescribed identical boundary conditions across all models. This included a heart rate of 70 bpm and a stroke volume of 73.5 mL with a prescribed flux during systole at the inflow plane and prescribed pressure during diastole (Fig. 1D). A volumetric flux was prescribed at the coronary arteries (Fig. 1D). After valve closure, a variable pressure was prescribed to mimic LV diastolic filling pressures. At the outflow plane, the 3D model was coupled to a 2-element Windkessel model to provide physiological pressure/flow boundary conditions (Fig. 1D) [23]. The Windkessel model had a resistance of 0.121 (Pa$\cdot$s)/mm$^3$ and a compliance of 9.37 mm$^3/$(Pa$\cdot$s). Each problem was run for 3 cardiac cycles, with a time step of 0.0008 seconds, to reach convergence. This was determined where the peak velocity and mean TPG were within 5% cycle to cycle.

The Navier Stokes solution was then used to solve for regions of high flow recirculation which is correlated with increased thrombosis risk [24]. To evaluate this, we calculated the residence time. This quantity was calculated as a field, detailing the amount of time a fluid particle spends inside any region in the aortic root and ascending aorta. To solve for the time a fluid particle has been in the region of interest ($\phi$), for any t $\in$ [0, T], we satisfy the following advection-diffusion equation [24]:

5 $$\begin{aligned}&\partial _{t}\phi +{\textbf {v}}\cdot \nabla \phi +\nabla \cdot {\varvec{D}}\nabla \phi ={\textbf {1}}, \,\qquad \qquad \qquad \text {on } \Omega , \end{aligned}$$

6 $$\begin{aligned}&\phi = 0, \qquad \qquad \qquad \qquad \qquad \qquad \qquad \qquad \qquad \,\, \text {on } \Gamma ^D, \end{aligned}$$

7 $$\begin{aligned}&{\varvec{D}}\nabla \phi \cdot \hat{{\textbf {n}}}=0, \qquad \qquad \,\,\,\qquad \qquad \qquad \qquad \quad \quad \, \text {on } \Gamma ^N, \end{aligned}$$

where ${\textbf {v}}$ is the predefined advective field solved from the Navier-Stokes equations and $\phi$=0 on the inflow and outflow faces ($\Gamma ^D$) with a Dirichlet boundary condition. The diffusivity (***D***) is set to 0 as it is assumed to have negligible impact. A constant source is added, making the field grow consistently with time, unless it is advected away and replaced by new fluid. We use the 3rd cycle solution from our fluids problem as the prescribed advective field. Each problem was run for 14 cardiac cycles so that there was less than a 5% change in model’s mean residence time. The field output was scaled by the total simulation time, T, which results in the relative RT field. This value varied from 0 to 1, where a value of 1 means that those blood particles do not leave the fluid volume, a value of 1/3 means it takes T/3 seconds for blood particles in that region to exit the domain, and a value of  0 means the blood particles leave the fluid volume nearly instantly.

### Quantitative Analysis

To quantify the impact of *Y-AAE*+SAVR vs SAVR we evaluated the following variables of interest:

1. Peak Velocity: This measure was calculated similar to echo measurements whereby we examined hemodynamics in the effective orifice area, taking the maximum velocity magnitude downstream of the aortic valve.
2. Mean TPG: This was defined as the average positive pressure difference between the inlet plane and a plane 30 mm downstream of the aortic valve during forward flow.
3. Relative RT (t/T): This was the amount of time (t) a particle has been in the 3D domain divided by the current time (T). We quantify the maximum and mean relative RT. To quantify if there are large volumes with high RT, we also evaluated the percentage volume that has a relative RT>0.2.

## Results

### *Y-AAE*+SAVR vs. SAVR Hemodynamics

**Fig. 2:**
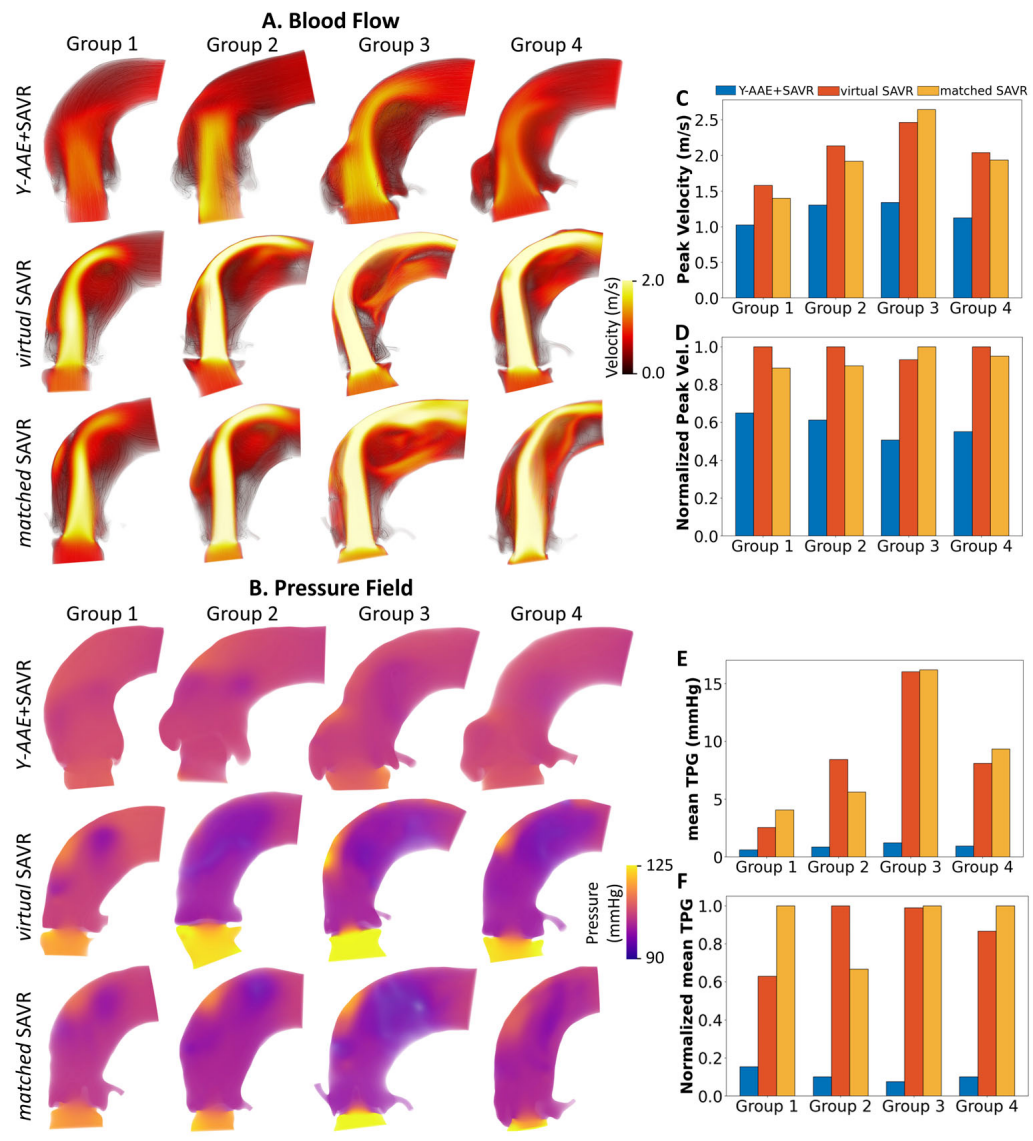
Pressure and flow results. Panel A visualizes the blood flow at peak velocity where streamlines and volume rendering are colored according to velocity magnitude. Results for (C) peak velocity and (D) peak velocity normalized by each group’s maximum peak velocity are shown. Panel B shows a volume rendering of the pressure field for all models at peak systole. Results across the cardiac cycle for the (E) mean TPG and (F) mean TPG normalized by each group’s maximum mean TPG. (*SAVR*, surgical aortic valve replacement; *TPG*, transcatheter pressure gradient; *Y-AAE*, Y-incision aortic annular enlargement)

Results for blood flow and pressure are presented by procedure type in Fig. 2: *Y-AAE*+SAVR (row one), *virtual*SAVR (row 2), and *matched*SAVR (row 3). For all groups, the *Y-AAE*+SAVR models demonstrated significantly lower velocities and a wider jet across the aortic valve. The peak velocities are shown in Fig. 2B. Figure. 2C shows the peak velocity for each model normalized by it’s group’s peak velocity, emphasizing percent decrease in peak velocity from a Y-AAE procedure. The *Y-AAE*+SAVR models had a 41.1% and 37.5% decrease in peak velocity compared to the *virtual*SAVR models *matched*SAVR models, respectively. SAVR only models (*virtual*SAVR and *matched*SAVR) showed consistency, with an average difference in peak velocity of 9%.

The pressure field at peak velocity is visualized in Fig. 2B. For the *Y-AAE*+SAVR models there appeared to be minimal pressure change across the aortic valve. For the *virtual*SAVR and *matched*SAVR models, there was noticeably higher pressure before the valve and large gradation across the valve. The mean TPG normalized by each group’s maximum mean TPG is shown in Fig. 2F. We observed a decrease in the *Y-AAE*+SAVR mean TPG of 86.5-87.9% compared to *virtual*SAVR and *matched*SAVR models. On average, the *virtual*SAVR and *matched*SAVR demonstrated a 25% difference in mean TPG. Results agreed with the trends shown in the TTE follow-up for these patients which is discussed further in the Supplementary Material (Fig. S1)

### *Y-AAE*+SAVR vs. SAVR Residence Time

**Fig. 3:**
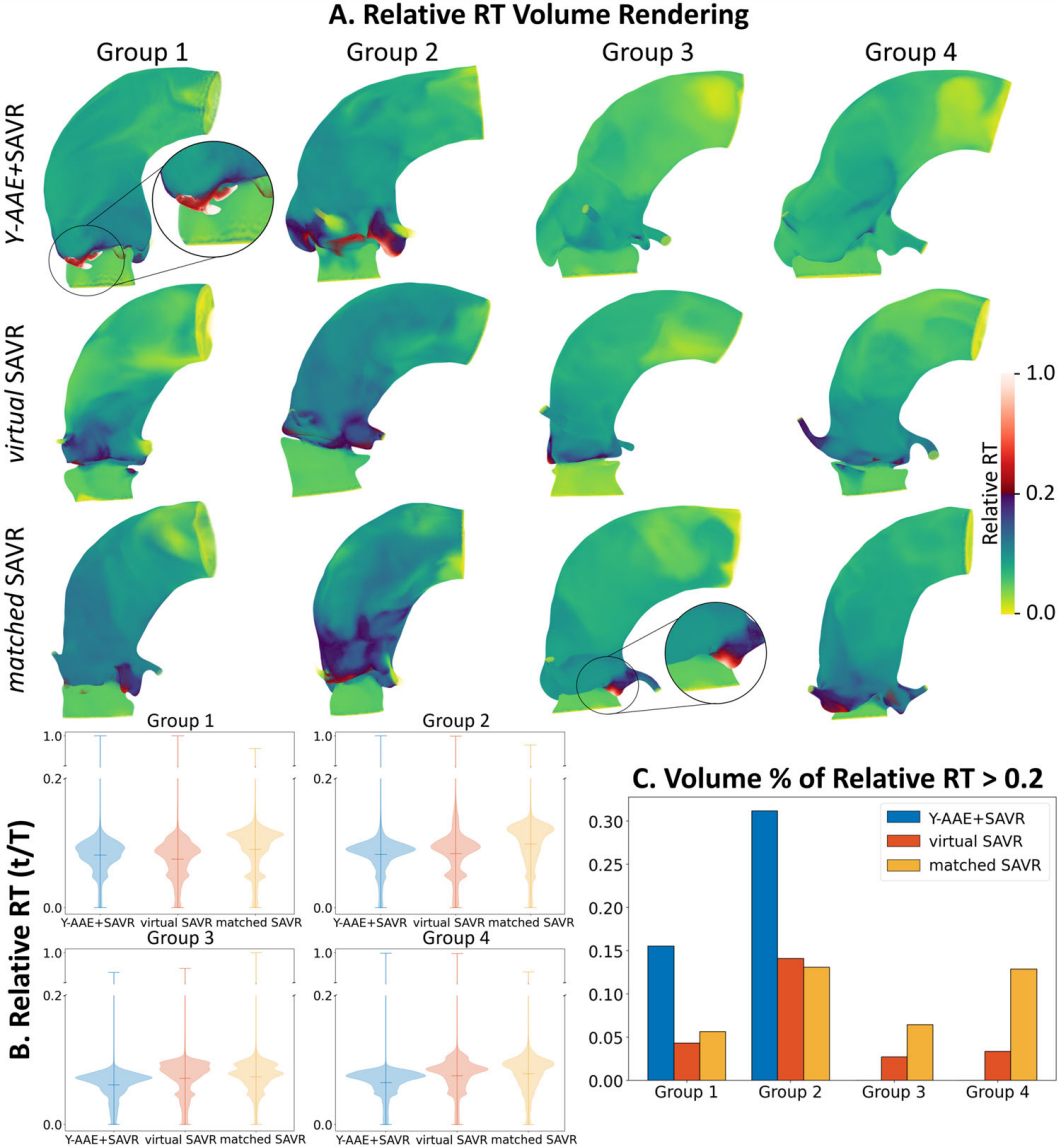
(A) Volume rendering of relative RT results. (B) Violin plots showing the relative RT distribution. There is a break in the y-axis to better visualize the distribution with a relative RT<0.2. (C) Bar graph showing the percent volume of relative RT>0.2. (RT, residence time; SAVR, surgical aortic valve replacement; Y-AAE, Y-incision aortic annular enlargement)

Results of the relative RT are shown in Fig. 3A. Regions of increased relative RT were found in the aortic sinuses, e.g., in Group 2 *Y-AAE*+SAVR and in Group 3 *matched*SAVR. The overall distribution of relative RT is shown in Fig. 3B. These show that while there were some regions with high RT, much of the blood volume has a relative RT less than 0.2, i.e., 80% of the blood volume had complete washout in  3 cardiac cycles. The *Y-AAE*+SAVR models had on average a 5.4% and 4.3% decrease in mean and maximum relative RT compared to the *virtual*SAVR models, respectively. Compared to the *matched*SAVR models, the *Y-AAE*+SAVR showed a 15.1% decrease in mean relative RT and a 33.3% increase in maximum relative RT.

The bar charts in Fig. 3C display the normalized volume of blood with relative RT greater than 0.2. For Groups 1 and 2 the *Y-AAE*+SAVR models contained a greater percentage of volume with higher relative RT. However, in Groups 3 and 4, the *Y-AAE*+SAVR models had less volume with a high relative RT compared to their control models (*virtual*SAVR and *matched*SAVR).

### The Impact of Y-AAE on Future TAVR-in-SAV

**Fig. 4:**
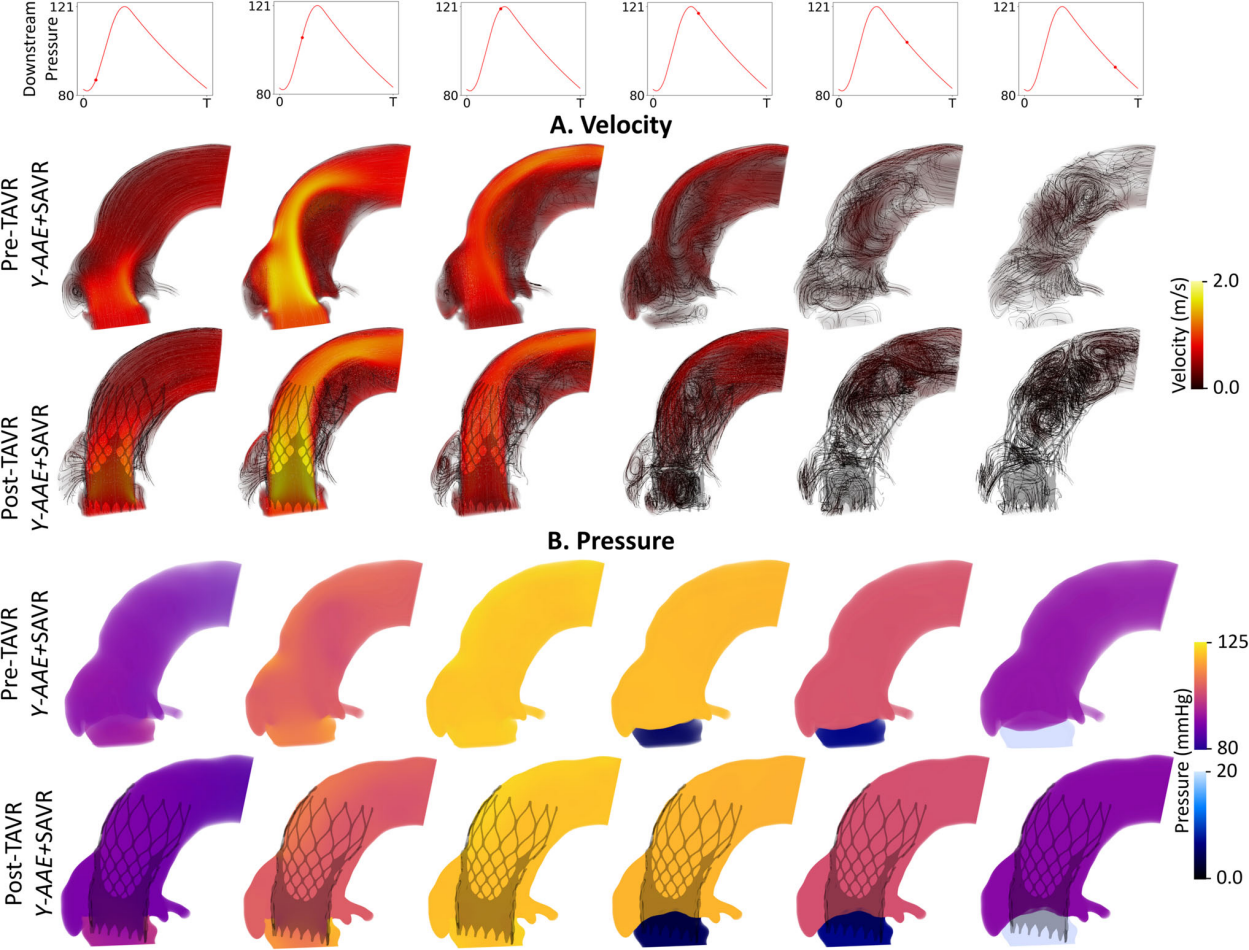
(A) Velocity and (B) pressure field for Group 3 Y-AAE+SAVR models, pre- and post-TAVR deployment, over the cardiac cycle. The top row shows the downstream pressure trace, and the red circle demarcates the progress in the cardiac cycle. Similar flow and pressure results for all models are shown in the Supplementary Material (Figs. S2, S3, S4, S5 and S6) and videos illustrating blood flow, pressure, and residence time over one cardiac cycle are also provided. (SAVR, surgical aortic valve replacement; TAVR, transcatheter aortic valve replacement; Y-AAE, Y-incision aortic annular enlargement)

To evaluate the impact of a Y-AAE for potential future TAVR-in-SAV, we compared the local hemodynamics between the pre- and post-TAVR state. Velocity and pressure fields were plotted across the cardiac cycle for Group 3 *Y-AAE*+SAVR pre- and post-TAVR. Figure 4A shows the pre- and post-TAVR velocity field over the cardiac cycle. During systole, we observed that the TAVR caused a funneling effect on the flow. During diastole, there was less circulation of fluid in the sinuses. Figure 4B displays the pre- and post-TAVR pressure field over the cardiac cycle. Even with the implanted TAVR, there is no visibly significant change in the TPG.

**Fig. 5:**
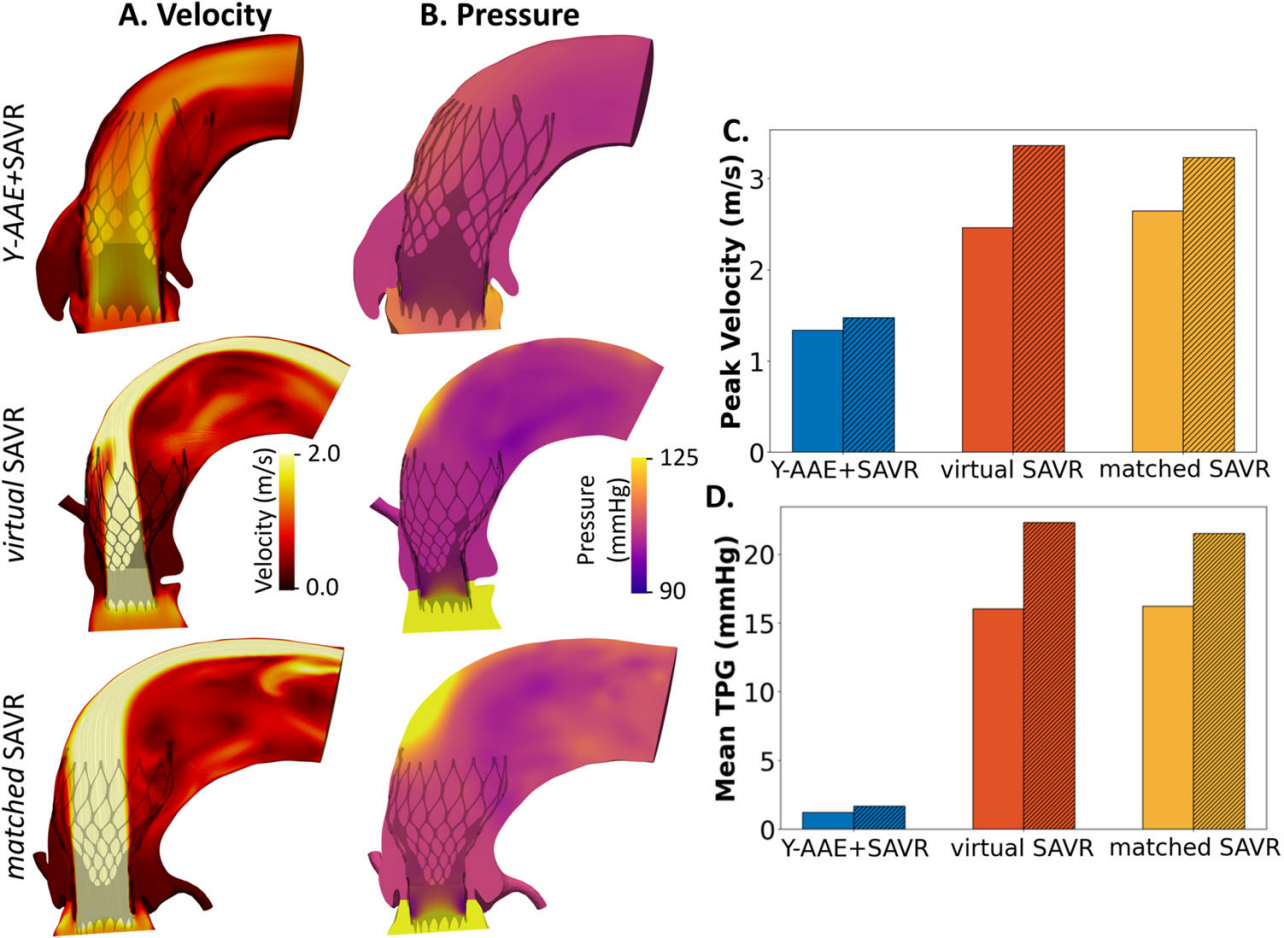
TAVR-in-SAVR results. (A) A cut region of the velocity field at peak systole colored by velocity magnitude. (B) A cut region of the pressure field at peak systole. (C) Peak velocity pre- (solid bars) and post-TAVR (hatched bars). (D) mean TPG at pre- and post-TAVR. (SAVR, surgical aortic valve replacement; TAVR, transcatheter aortic valve replacement; TPG, transcatheter pressure gradient; Y-AAE, Y-incision aortic annular enlargement)

The velocity and pressure fields at peak systole are shown in Fig. 5. The deployment of the TAVR decreased the GOA by 17.2%, 23.3% and 20.7% for the *Y-AAE*+SAVR model, *virtual*SAVR model, and *matched*SAVR model, respectively. This led to an average increase in peak velocity of 23.1%, and in mean TPG by 36.8%. This is summarized with the bar graphs in Figs. 5B,C. The TAVR-in-SAVR *Y-AAE*+SAVR model had a 55.1% lower peak velocity and a 92% lower mean TPG compared to the *virtual*SAVR and *matched*SAVR TAVR-in-SAVR models.

**Fig. 6:**
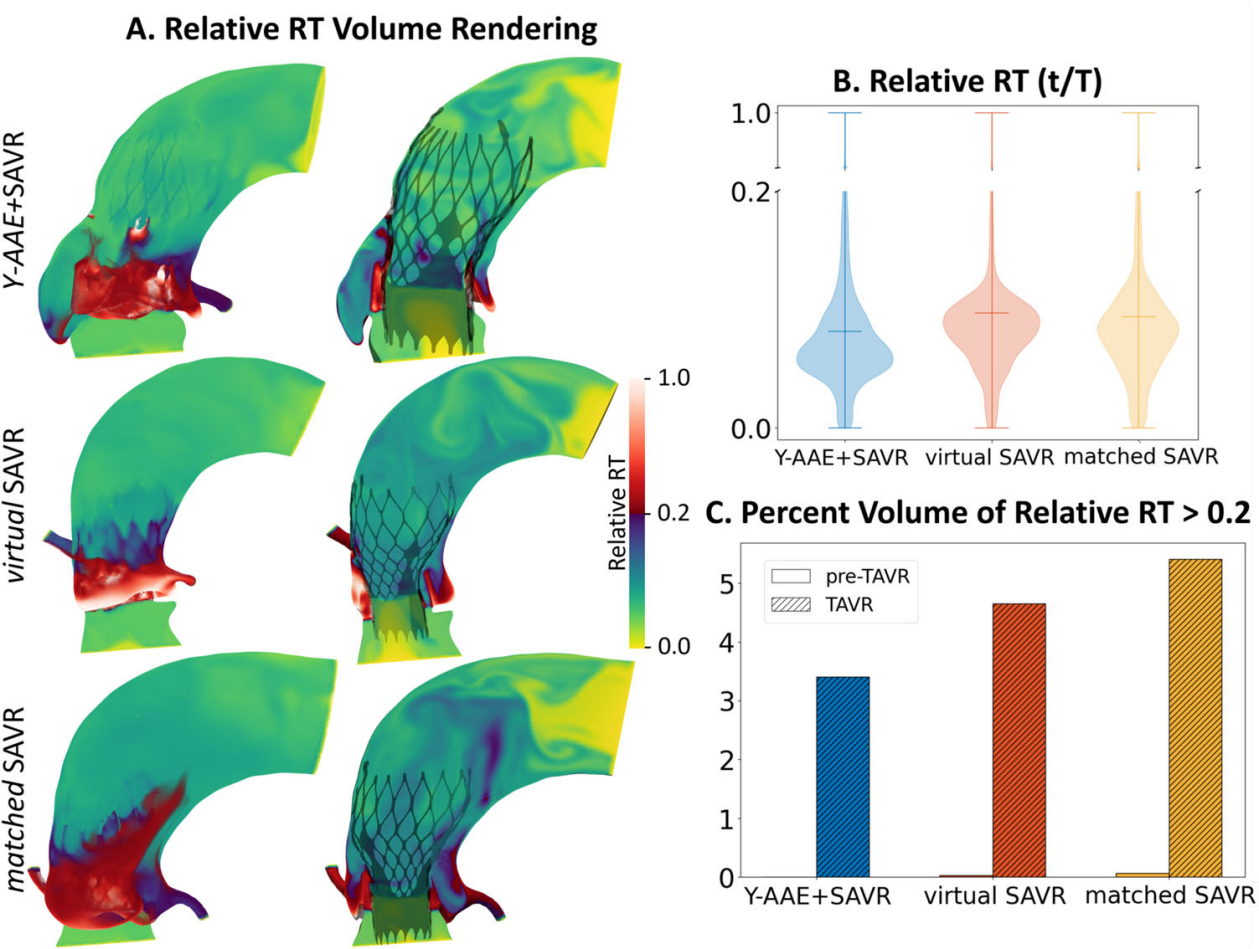
(A) Visualization of relative RT with volume rendering (left) and cut region (right) for the TAVR-in-SAVR models. (B) Violin plot of relative RT with a break in the y-axis at 0.2. (C) Bar graph showing the percent volume of relative RT>0.2 pre- (solid) and post-TAVR (hatched). (RT, residence time; SAVR, surgical aortic valve replacement; TAVR, transcatheter aortic valve replacement; Y-AAE, Y-incision aortic annular enlargement)

There was a stark increase in the RT in the TAVR-in-SAVR models, especially in the neo-sinuses and the anatomic sinus (Fig. 6A). Compared to the pre-TAVR state, the normalized blood volume with relative RT higher than 0.2 increased significantly, going from less than 0.0001% to 3.4% for the *Y-AAE*+SAVR models, 0.02% to 4.6% for the *virtual*SAVR, and going from 0.06% to 5.4% for the *matched*SAVR model (Fig. 6B). Likewise, the mean RT increased for all models on average by 31% due to the TAVR deployment.

## Discussion

In this study, we developed a cohort of SAVR patients with and without aortic annular enlargement to compare local hemodynamics and isolate the impact of the Y-AAE procedure. Additionally, we used the preoperative Y-AAE patient anatomy and virtually implanted a SAVR to serve as another control model. To focus on the influence of the surgical intervention and control for inter-patient variability, we prescribe identical boundary conditions across all models. This approach allowed us to attribute observed changes in hemodynamic outcomes-such as flow velocity and pressure distribution-more directly to the intervention rather than to patient-specific differences in the cardiovascular system. CFD modeling also enabled us to evaluate RT in the models to identify differences in flow recirculation and potential for thrombosis. Finally, we examined the impact of TAVR-in-SAVR on the hemodynamics for patients with and without enlargement.

*Y-AAE*+SAVR models showed a reduction in transvalvular pressure gradients across the aortic valve compared to *virtual*SAVR and *matched*SAVR models. This suggests that *Y-AAE*+SAVR could provide a smoother transition for blood flow, reduce the workload on the heart, and potentially leading to better patient outcomes [7, 9, 25]. Importantly, the mean TPG decreased significantly in the *Y-AAE*+SAVR models, which is indicative of improved hemodynamic efficiency and a reduction in the risk of PPM, translating into clinical benefits, such as reduced symptoms and improved cardiac function [9, 26, 27]. This aligns with the results from Rodes-Cabau et al., who concluded that in patients with a small, pre-operative aortic annulus size, larger post-operative valve area resulted in better hemodynamics and outcomes [27]. Furthermore, the success of the SAVR with Y-AAE procedure in maintaining low post-operative TPG should also significantly reduce the risk of surgical valve deterioration (SVD), particularly in younger patients, as demonstrated by a study conducted by Johnston et al [28]. Results showed that *Y-AAE*+SAVR models exhibited significantly lower velocities and a wider jet across the aortic valve during systole compared to *virtual*SAVR and *matched*SAVR models. This was expected as previous studies show an inverse relationship between aortic valve area and peak velocity [29]. Furthermore, the Y-AAE procedure was an extremely effective method to reduce the peak velocity across the valve, which helps in reducing shear stress and potential damage to blood components [8].

The analysis of RT revealed no significant differences between the 3 model types (*Y-AAE*+SAVR, *virtual*SAVR, and *matched*SAVR). The *Y-AAE*+SAVR procedure, on average, showed a decrease in mean RT compared to the *virtual*SAVR and *matched*SAVR models. However, there was no significant difference between models in maximum RT or the volume percent with high RT. We found that regions of high RT formed where the anatomy was more complex and formed small pockets of fluid, making it more difficult for blood washout. This did not depend on the Y-AAE procedure. This explains why the distribution of RT showed that in certain groups (Groups 1 & 2), *Y-AAE*+SAVR models had more volume with higher RT, while in other groups (Groups 3 & 4), they had less. This aligns with previous findings that found that thrombus formation is sensitive to patient’s aortic root morphology’s interaction with the implanted valve [30]. Examples of this can be seen in the zoomed in regions displayed in Fig. 3. This variability highlights the importance of patient-specific morphology and that the Y-AAE procedure does not increase the amount of blood recirculation and therefore thrombus formation. This is further supported by follow-up echocardiograms, which over a period of up to 3 years, show no thrombosis on the patch or bioprosthetic valve in the *Y-AAE*+SAVR patients [9].

To evaluate the impact of a Y-AAE on potential future TAVR-in-SAVR we virtually deployed a CoreValve into the *Y-AAE*+SAVR, *virtual*SAVR, and *matched*SAVR models of Group 3. These results showed that TAVR deployment increased peak velocities and pressure gradients across all models, as expected [31]. Interestingly, however, the *Y-AAE*+SAVR TAVR-in-SAVR models exhibited lower peak velocity and mean TPG compared to the pre-TAVR control models (vitrualSAVR and *matched*SAVR). Based on previous findings of Kherallah et al., the *Y-AAE*+SAVR model’s TAVR-in-SAVR mean TPG value would also suggest a lower risk of one-year all-cause mortality, stroke, myocardial infarction, and reintervention [32]. This highlights a significant advantage of the Y-AAE procedure for potential TAVR-in-SAVR applications, particularly relevant in younger patients with SAVR. Like the pre-TAVR state, the *Y-AAE*+SAVR model did not exhibit greater blood stasis than the *matched*SAVR or *virtual*SAVR control models. The study also observed a significant increase in RT in the sinuses post-TAVR deployment-namely, in the neo-sinuses and the anatomic sinus, in support with previous literature indicating the importance of blood-skirt interactions in thrombogenesis [30, 33]. The normalized volume of RT>0.2 increased markedly, indicating potential areas of concern for blood stasis and thrombus formation post-TAVR deployment. The mean RT also increased by an average of 31% across all models and the maximum relative RT was 1.0 for all models, meaning there was not complete blood washout.

### Limitations

There were a few limitations to this study that should be mentioned. To enable like-for-like comparisons, the study utilized a static anatomical domain for the simulations, rather than modeling aortic movement. This static approach likely represents a worst-case scenario for RT, as the natural pulsatile motion of the aorta should induce more fluid circulation and better washout. This likely has limited impact on the velocity and pressure metrics; however, which were driven by the valve orifice. While prescribing the same boundary conditions across all models enhances the interpretability of our results, we acknowledge that our analysis was limited to a single set of hemodynamic conditions-namely, fixed stroke volume and heart rate. In reality, patient-specific variations and physiological states such as exercise can significantly influence hemodynamic behavior, and future studies could explore how surgical outcomes vary under different loading conditions. Furthermore, since the aortic valve opening and closure occurs extremely quickly, the valve was modeled as an opening/closing orifice plane consistent with manufacture measures [9]. This simplification represents the best-case scenario for the peak velocity and mean TPG, as the presence of leaflets will reduce this orifice area. Consequently, the addition of leaflet dynamics will likely only further exacerbate differences observed between Y-AAE and non-AAE models. Additionally, this work assumed that residence time is a valuable surrogate for thrombosis potential [34]. However, it is known that flow stasis and the shear experienced by blood both play a critical role in eventual thrombus formation and could be a factor to consider in future studies. Lastly, while this study focused on the hemodynamics of enlargement, the mechanics of the aortic root wall and potential change to internal root stresses due to enlargement present an important area for further investigation.

### Conclusion

To conclude, this study examined the hemodynamic impact of Y-incision AAE in SAVR. The results demonstrated that *Y-AAE*+SAVR significantly reduced peak flow velocities (39.3% decrease) and mean transvalvular pressure gradients (87.2% decrease), leading to improved hemodynamic performance compared to SAVR alone. Furthermore, *Y-AAE*+SAVR led to a 10.3% reduction in mean RT, with no consistent trend observed in maximum RT, indicating no additional risk of significant thrombus formation. Additionally, the Y-AAE procedure showed a significant hemodynamic advantage in simulated valve-in-valve scenarios, with a 55% lower peak velocity and a 92% lower mean TPG, suggesting that the Y-AAE procedure is beneficial for future TAVR-in-SAVR applications. The findings highlight the importance of Y-AAE in enhancing hemodynamic efficiency and reducing the risk of patient-prosthesis mismatch, thus improving patient outcomes post-SAVR, and should be utilized in SAVR frequently.

## Clinical Relevance

These findings have important clinical implications for surgical planning in patients with severe aortic stenosis. By demonstrating that Y-AAE improves hemodynamics and reduces the risk of prosthesis-patient mismatch, this work supports its consideration, particularly in patients who may require future valve-in-valve interventions. Furthermore, the reduction in transvalvular gradients, without a significant increase in residence time, suggests that Y-AAE could enhance both short- and long-term outcomes without elevating thrombosis risk. Ultimately, this study provides mechanistic evidence to inform clinical decision-making regarding annular enlargement during SAVR procedures.

## Supplementary Information

Below is the link to the electronic supplementary material.

Supplementary file 1 (pdf 1172 KB)

## Data Availability

The data that support the findings of this study are available from the corresponding author upon reasonable request.

## Abbreviations

AS: Aortic Stenosis
BPM: Beats Per Minute
CFD: Computational Fluid Dynamics
CT: Computed Tomography
GOA: Geometric Orifice Area
PPM: patient-prosthesis mismatch
RT: residence time
SAVR: Surgical Aortic Valve Replacement
SVD: Surgical Valve Deterioration
t: current time
T: total simulation time
TAVR: Transcatheter Aortic Valve Replacement
TPG: Transvalvular Pressure Gradient
Y-AAE: Y-incision Aortic Annular Enlargement
