# Supplementary material for: Modeling the Hemodynamic Impact of Y-incision Aortic Annular Enlargements on Aortic Valve Replacement and Valve-in-Valve Procedures
Source: J Cardiovasc Transl Res. 2025 Jun 11;18(4):876–87. doi: 10.1007/s12265-025-10634-x (PMC12436585; doi:10.1007/s12265-025-10634-x)
Supplement: Supplementary file 1 — (pdf 1172 KB) [file 12265_2025_10634_MOESM1_ESM.pdf]

Modeling the Hemodynamic Impact of Y-incision Aortic Annular Enlargements on Aortic Valve Replacement and Valve-in-Valve Procedures

Journal of Cardiovascular and Translational Research

Mia Bonini[1*], Surya Sanjay[1*], Maximilian Balmus[2], Alexander Makkinejad[3], Katelyn Monaghan,[3] Marc Hirschvogel[4], Nicholas Burris, MD[5], Bo Yang, MD[3], and David Nordsletten[1,2,3]

[1]Department of Biomedical Engineering, University of Michigan, Ann Arbor, MI, USA
[2]Department of Biomedical Engineering and Imaging Sciences, King's College London, London, UK
[3]Department of Cardiac Surgery, Michigan Medicine, Ann Arbor, MI, USA
[4]MOX, Dipartimento di Matematica, Politecnico di Milano, Milan, Italy
[5]Department of Radiology, University of Michigan, Ann Arbor, MI, USA
[*]These authors contributed equally to this manuscript.

Corresponding Authors:
Mia Bonini
Emails: mbonini@umich.edu

Table S1: Patient Data

	**Group 1**		**Group 2**		**Group 3**		**Group 4**	
	Y-AAE Patient A	***Matched Patient B***	***Y-AAE Patient A***	***Matched Patient B***	***Y-AAE Patient A***	***Matched Patient B***	***Y-AAE Patient A***	***Matched Patient B***
**Age (y/o)**	65	70	65	63	70	64	67	55
**Sex**	M	M	F	F	F	F	F	F
**EF (%)**	55	65	65	65	65	75	60	55
**BSA**	2.2	2.12	2.23	2	2.17	1.92	2.29	1.89
**Native Annulus Diameter (mm)**	25	25	23	21	21	21	23	23
**SAVR Size (mm)**	29	25	29	23	27	21	29	23

Patient *Outcomes*
Follow up echocardiograms were performed for the patients in this study (*Y-AAE*+SAVR and *matched*SAVR). It is important to note that in our computational study we applied the same boundary conditions (CO, HR, etc.) to all models to isolate the impact of the patient anatomy. In reality, each patient's unique heart function will influence their hemodynamics (mean TPG, peak velocity, etc.). Therefore, our

computational results will not match the patient's echocardiogram results. However, the echo data verifies that an Y-AAE leads to significantly lower mean transvalvular pressure gradients and larger aortic valve areas (Figure S1). Furthermore, the echocardiogram data also shows there was no thrombus around the large valves.

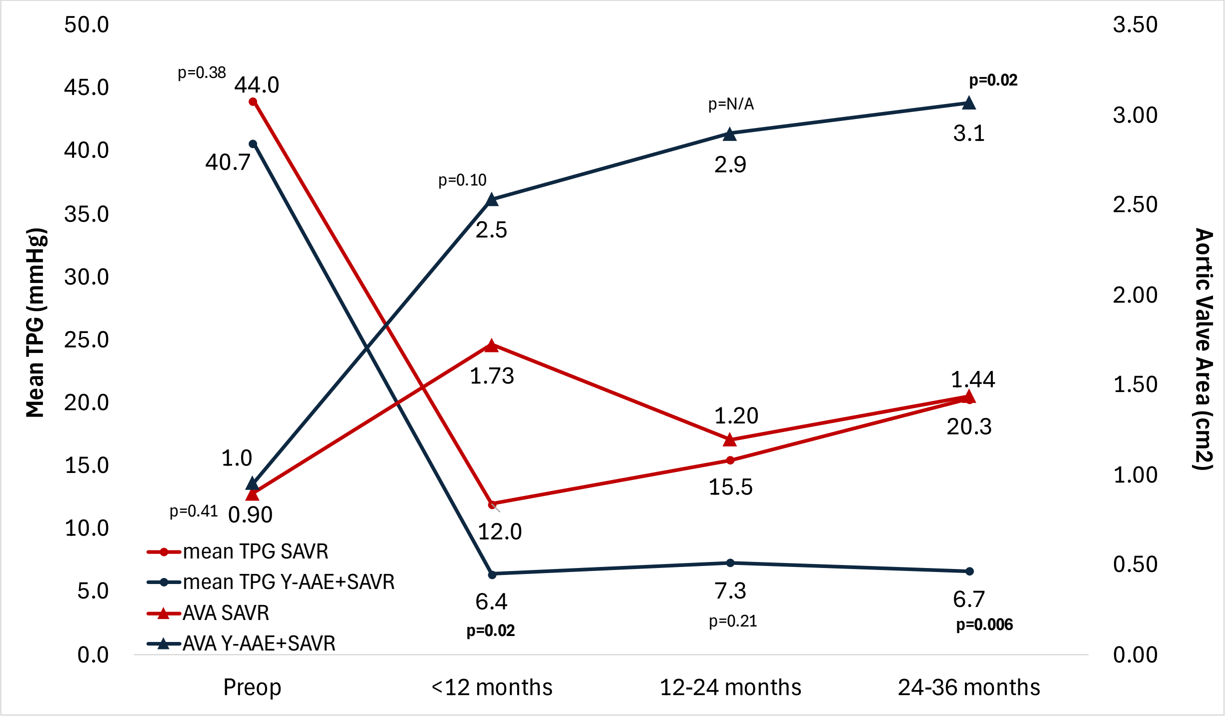

Figure S1: Echocardiogram results reporting changes in estimated aortic valve area and mean transvalvular pressure gradient over time following Y-incision aortic annular enlargement in patients with severe aortic stenosis as the primary indication of surgery. *AVA*, Aortic valve area; *SAVR,* surgical aortic valve replacement; *TPG*, transvalvular pressure gradient; *Y-AAE,* Y-incision aortic annular enlargement

Computational Results

The following figures display the velocity, pressure, and relative residence time over one cardiac cycle for all groups.

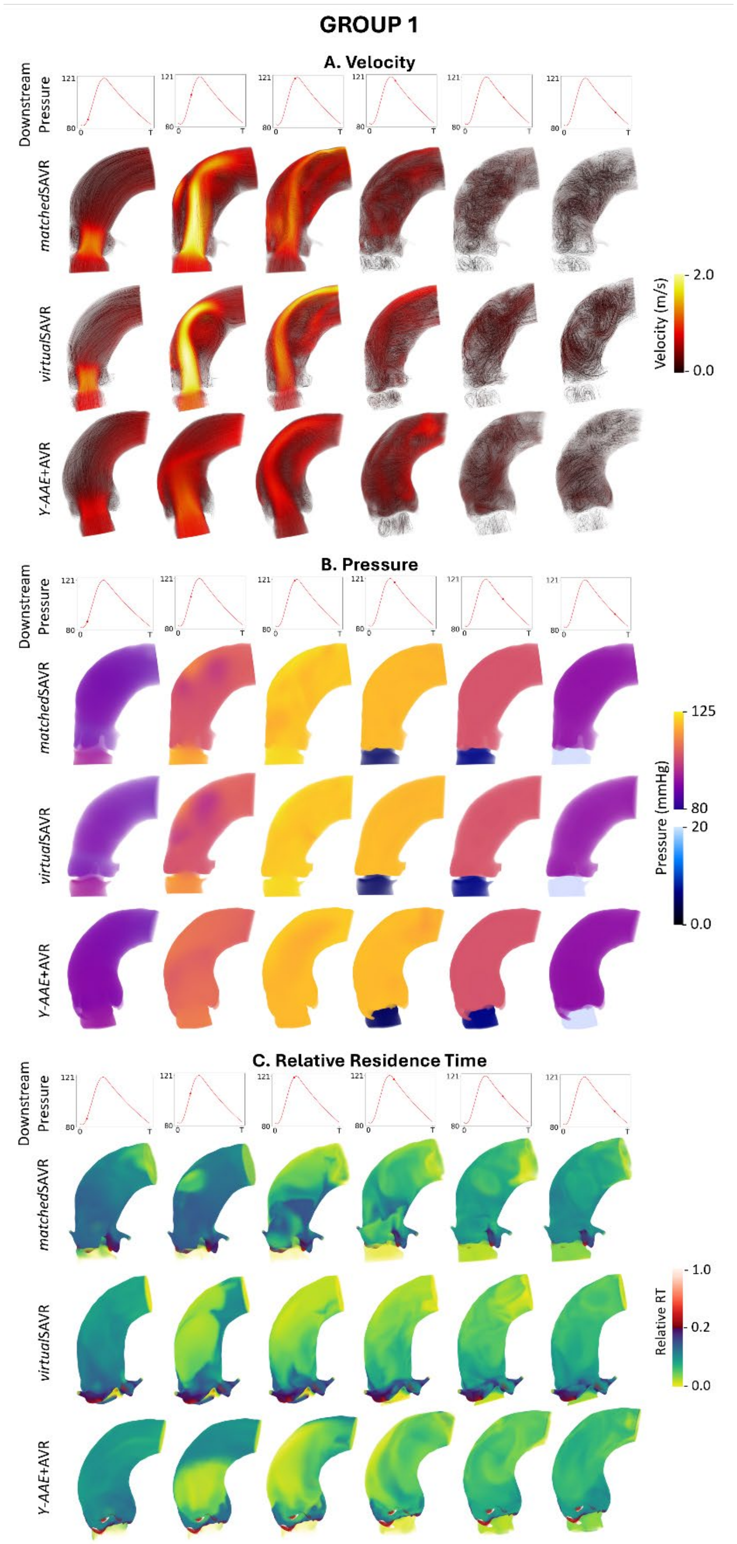

Figure S2: A) Velocity fields, B) pressure fields, and C) relative residence time field over the cardiac cycle for Group 1 (*Y-AAE*+SAVR, *virtual*SAVR, *matched*SAVR)

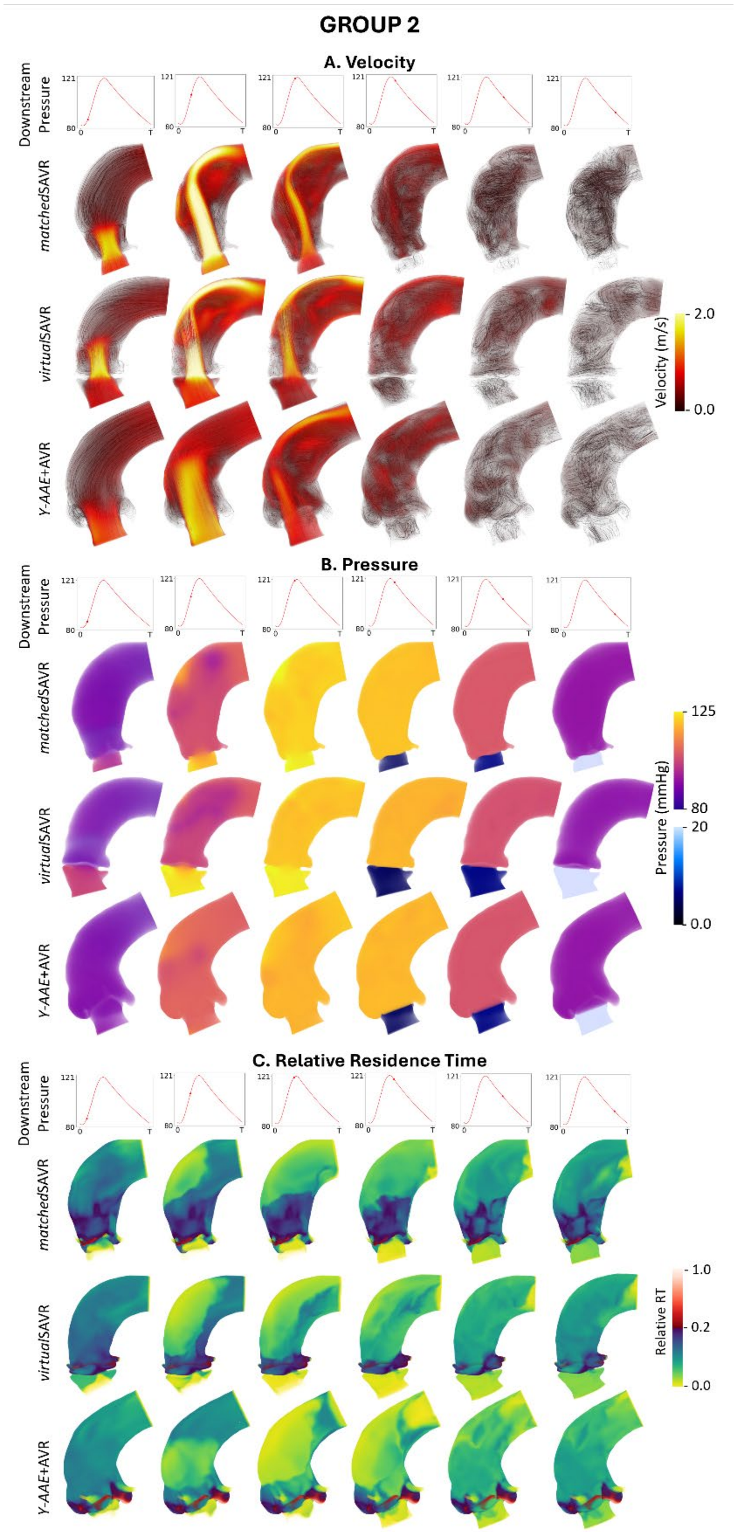

Figure S3: A) Velocity fields, B) pressure fields, and C) relative residence time field over the cardiac cycle for Group 2 (*Y-AAE*+SAVR, *virtual*SAVR, *matched*SAVR)

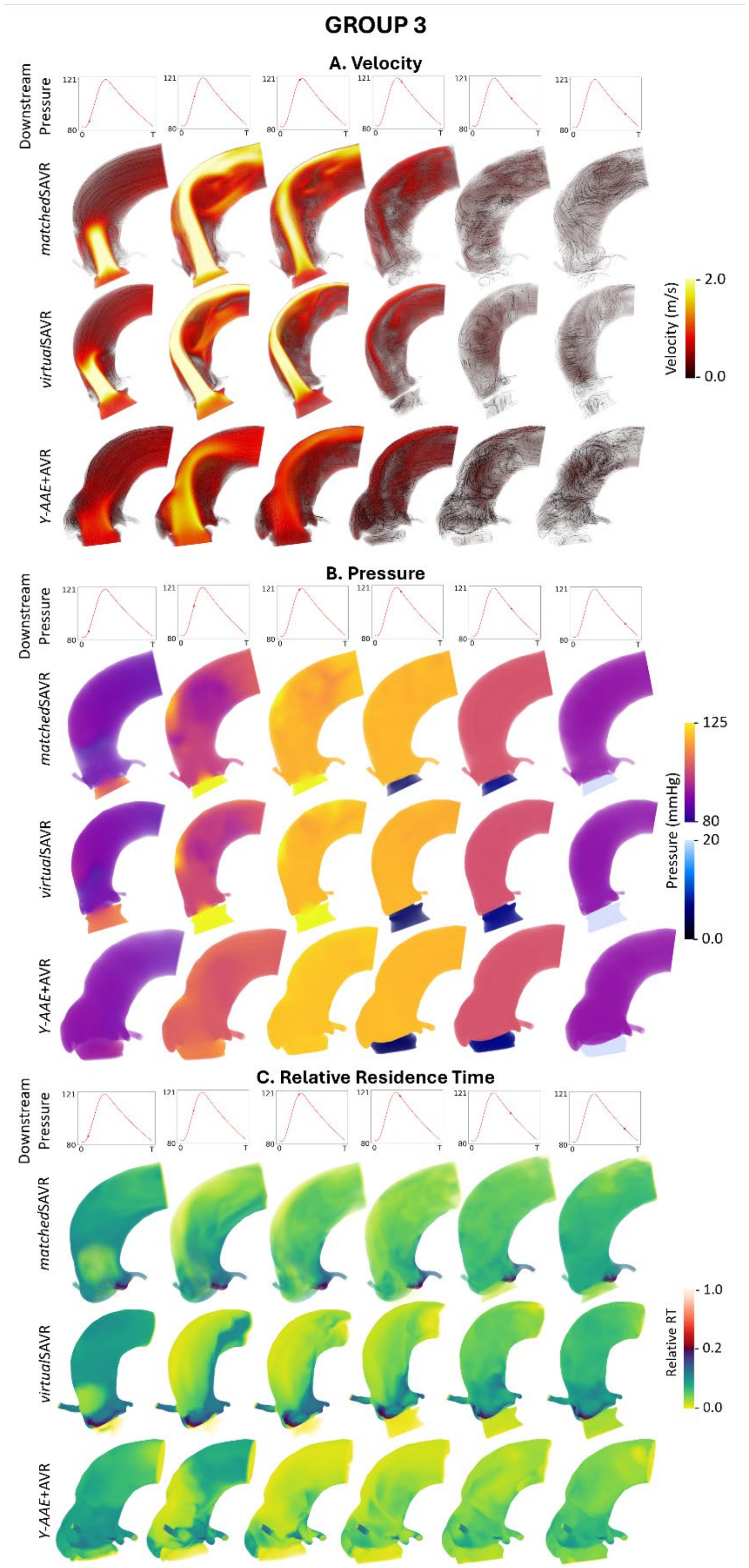

Figure S4: A) Velocity fields, B) pressure fields, and C) relative residence time field over the cardiac cycle for Group 3 (*Y-AAE*+SAVR, *virtual*SAVR, *matched*SAVR)

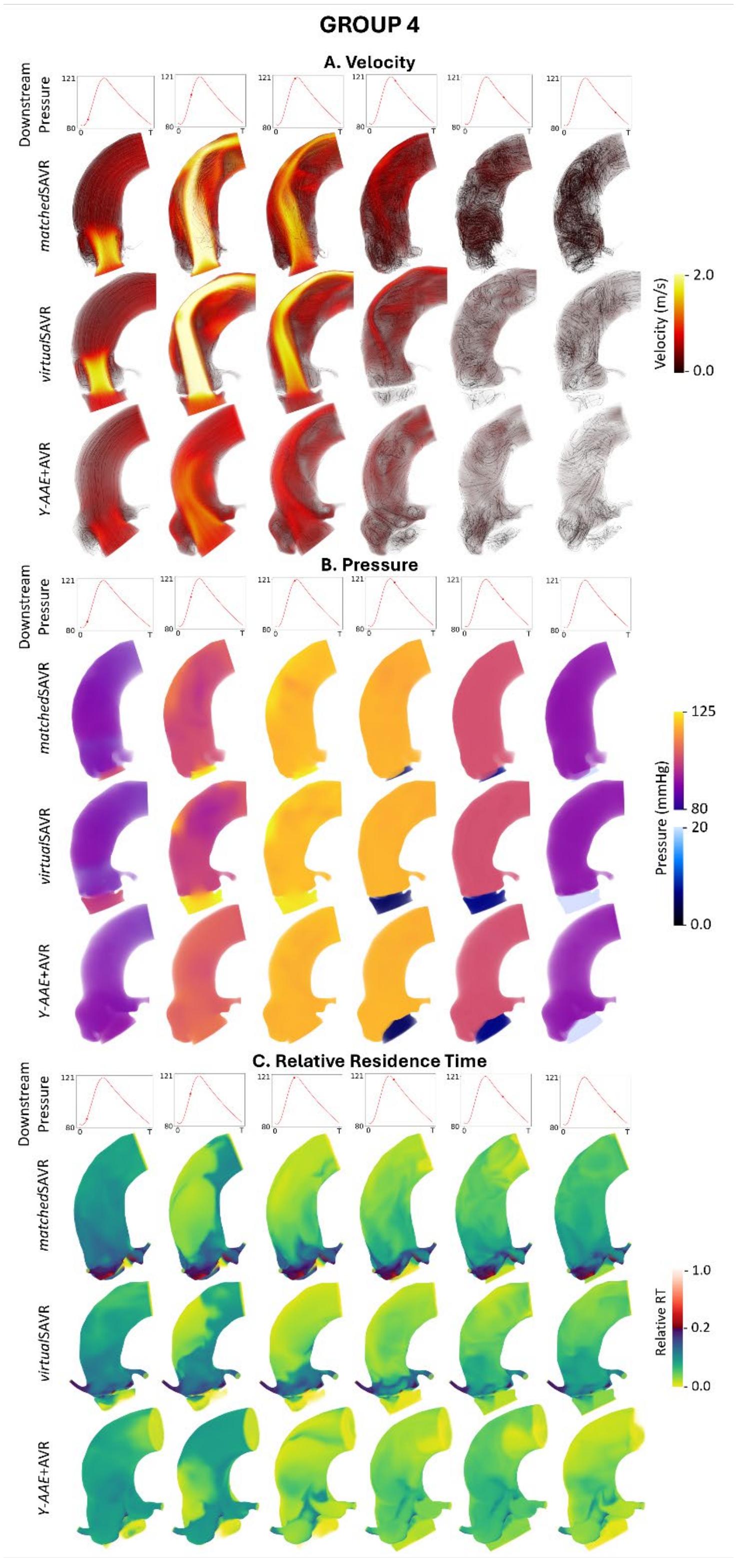

Figure S5: A) Velocity fields, B) pressure fields, and C) relative residence time field over the cardiac cycle for Group 4 (*Y-AAE*+SAVR, *virtual*SAVR, *matched*SAVR)

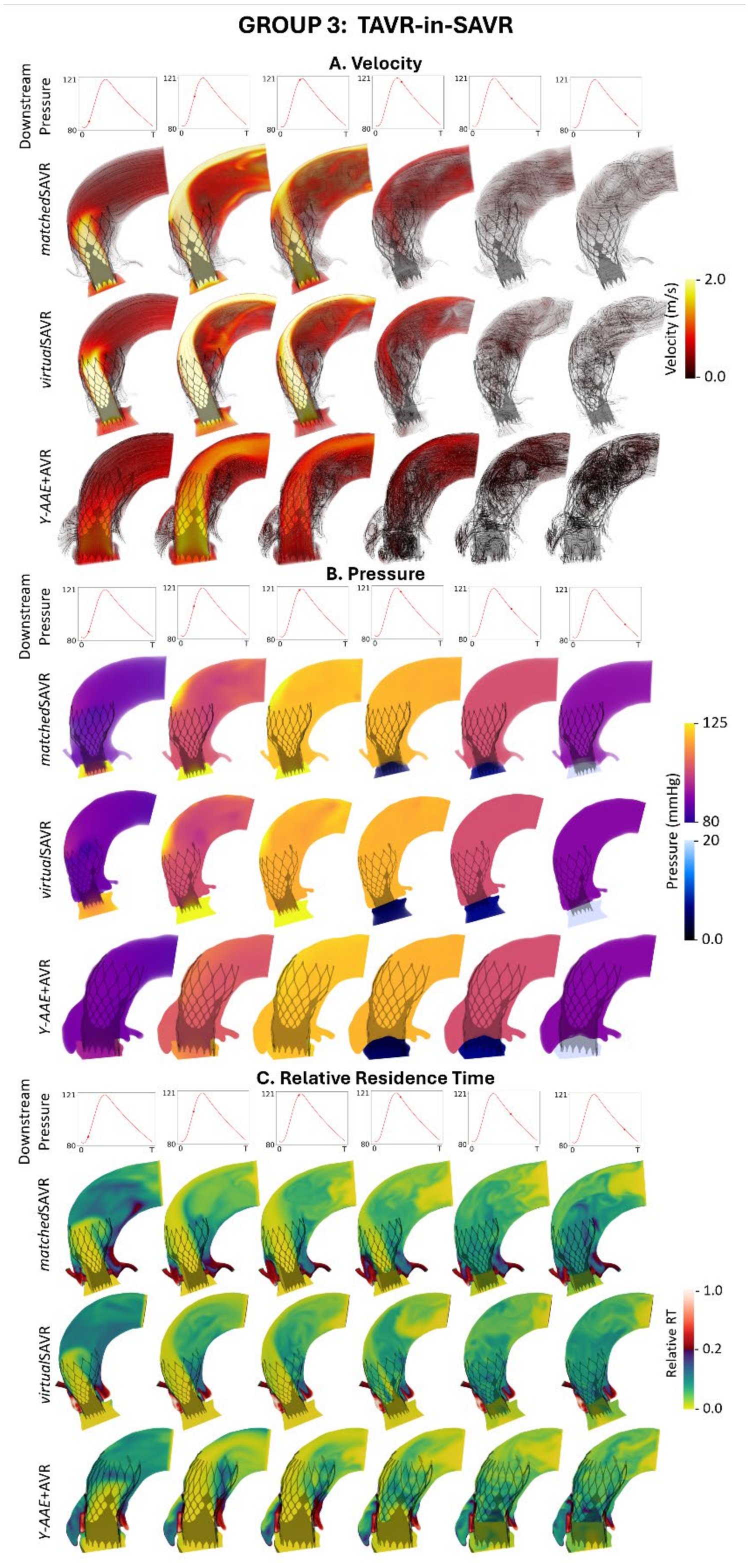

Figure S6: A) Velocity fields, B) pressure fields, and C) relative residence time field over the cardiac cycle for Group 3 TAVR-in-SAVR (*Y-AAE*+SAVR, *virtual*SAVR, *matched*SAVR)
